# Camera Traps Can Be Heard and Seen by Animals

**DOI:** 10.1371/journal.pone.0110832

**Published:** 2014-10-29

**Authors:** Paul D. Meek, Guy-Anthony Ballard, Peter J. S. Fleming, Michael Schaefer, Warwick Williams, Greg Falzon

**Affiliations:** 1 Invasive Animals CRC, Coffs Harbour, New South Wales, Australia; 2 Vertebrate Pest Research Unit, NSW Department Primary Industries, Armidale, New South Wales, Australia; 3 School of Environmental and Rural Sciences, University of New England, Armidale, New South Wales, Australia; 4 Vertebrate Pest Research Unit, NSW Department Primary Industries, Orange, New South Wales, Australia; 5 School of Science and Technology, University of New England, Armidale, New South Wales, Australia; 6 Marine and Atmospheric Research, CSIRO, Canberra, Australian Capital Territory, Australia; 7 National Acoustic Laboratory, Sydney, New South Wales, Australia; Institute of Zoology, China

## Abstract

Camera traps are electrical instruments that emit sounds and light. In recent decades they have become a tool of choice in wildlife research and monitoring. The variability between camera trap models and the methods used are considerable, and little is known about how animals respond to camera trap emissions. It has been reported that some animals show a response to camera traps, and in research this is often undesirable so it is important to understand why the animals are disturbed. We conducted laboratory based investigations to test the audio and infrared optical outputs of 12 camera trap models. Camera traps were measured for audio outputs in an anechoic chamber; we also measured ultrasonic (n = 5) and infrared illumination outputs (n = 7) of a subset of the camera trap models. We then compared the perceptive hearing range (n = 21) and assessed the vision ranges (n = 3) of mammals species (where data existed) to determine if animals can see and hear camera traps. We report that camera traps produce sounds that are well within the perceptive range of most mammals’ hearing and produce illumination that can be seen by many species.

## Introduction

Camera traps are being used widely throughout the world although the limitations and constraints of these devices are rarely considered. The study of animal ecology, biology and behaviour requires thorough planning, robust analysis and an element of good luck. Irrespective of the tools being used, there will always be expected errors, variability, unknowns or biases, described as being similar to the “Observer Effect” or “Heisenberg’s Uncertainty Principle” [1]. The study of animals can only provide an insight into their life history; nothing is absolute and understanding the variability is an important component of research investigations. Camera trapping is a survey tool that has improved our capacity to infer the life history of animals, especially where minimising observer effects on animal behaviour is critical [2]–[4]. Some consider that camera traps are a non-intrusive method of studying animals [5]. However, there is increasing evidence throughout the world that animal behaviour is affected by the presence of camera traps [6]–[9]. In some circumstances this ‘effect’ may have little impact on the investigation. In other studies, for example those using indices and mark-recapture estimators (e.g., [1], [10], [11]), it is paramount that the technology used does not alter animal behaviour during or between monitoring sessions to ensure constancy of detectability [8]. Where bias occurs, it is crucial that this effect is understood and measured when interpreting the results of the observations; “the accuracy of an index is irrelevant; precision is paramount” [11]. Irrespective of the hypothesis being tested, the effect on behaviour can scarcely be considered non-intrusive [8] if animals display behavioural responses to sampling tools.

Observations of responses to mensurative devices strongly imply that learning can occur as a consequence of exposure to the devices. For examples, camera traps could be detected by animals for the following reasons:

1. Auditory – by the emission of sounds from the electronic and mechanical components of the device: these could be in the infra, audible and ultra-sound ranges.
2. Olfactory – metal, plastic and human scents on the device [6], [9],
3. Learned association – avoidance of the camera trap through wariness of human presence at a site [6] or attraction to the camera trap through lures and food baits,
4. Visual (day) – neophobia towards foreign objects introduced into their environment; regular-shaped objects (essentially rectangular prisms) attached to trees or posts [12], [13],
5. Visual (night) – the flash of xenon light, white LED or infrared LED illumination [7].

The hearing and vision [22] of animals varies depending on their life history, hunting *modis operandi*, body size [23], [24] and favoured prey [25]. It is commonly accepted that the combination of hearing and vision is important for animal localisation acuity [22], for hunting and social interactions and to avoid predators [26].

### Auditory ranges

Hearing ranges are broad in mammals, as an example; mice (*Mus domesticus*) have a range from 2.3–92 kHz [29], horses (*Equus cabalus*) hear up to 33.5 kHz, cows (*Bos taurus*) to 35 kHz [32], kangaroo rat (*Dipodomys merriami*) to 74 kHz, while the rabbit (*Oryctologus cuniculus*) can only hear to 49 kHz., cotton rat (*Sugmondon hispidus*) to 72 kHz [29], wood rat (*Neotoma floridana*) to 56 kHz, grasshopper mouse (*Onychomys leucogaster*) 69 KhZ [33], and fox squirrel (*Sciurus niger*) 49 kHz [34]. A small Australian predator, the northern quoll (*Dasyurus hallucatus*) hear best from 8–10 kHz although their hearing range is 0.5–40 kHz [31]. Six Australian Brush-tailed possum (*Trichosurus vulpecula*) were trained to respond to frequencies of 88 kHz [35]. Only bats, dolphins and shrews have been reported to recognise and detect high frequency signals [36], although the authors propose that “it is not impossible that all primitive mammals are capable of echolocation”.

Our associated research primarily focuses on the management of introduced predators [1], wild dogs (*Canis lupus ssp*) and European red foxes (*Vulpes vulpes*) and to a lesser extent on feral cats (*Felis catus*). Feral and domestic cats have one of the broadest hearing ranges of all mammals [27], ranging from 48 Hz to 85 kHz, although responses have been reported up to 100 kHz [28]. Dogs show variability in sensitivity to sound depending on breed (6–45 kHz) (https://www.lsu.edu/deafness/HearingRange.html accessed 3 July 2013) and as high as 65 kHz [28], although this has been disputed [30]. Foxes have evolved with a wide ranging hearing capacity (0.9–34 kHz) with optimal hearing at 10–14 kHz and an upper limit of 34 kHz [25] and 65 kHz [28].

### Visual ranges

Dogs are known to have dichromatic colour vision with an upper limit of detection around 555 nm [16], while Mustelids have been reported to have the capacity to detect infrared light up to 870 nm [17]. In the case of Australian marsupials there is clear evidence of colour vision [18]–[20] with taxa variability in regards to spectral sensitivity (dichromatic vs trichromatic) [21].

Camera traps that use xenon white flash to illuminate animals have been widely used in hunting and wildlife research [7] even though there is concern that the bright flash affects the short and long term behaviour of target animals. In a study of Kinkajous (*Potos flavus*) behavioural avoidance of ‘canopy-highway’ branches where white flash camera traps were placed has been reported [8]. Tiger (*Panthera tigris tigris*) capture rates in Nepal decreased by 50% over 5 nights of camera trapping using xenon flash devices [7] and similar concerns have been raised in studies of grey wolves (*Canis lupus*) [14]. Technological advances have resulted in infrared camera traps dominating the market based on claims that animals can’t see the infrared flash [15].

Most of the mammal species being studied using camera traps are nocturnal-crepuscular animals, although not always [19], with some showing a slight preponderance for diurnal activity; so their eye physiology reflects this behaviour. It would not be accurate to state that animals can “see in the dark”; a more accurate description may be that they are able to “see what is in the dark” [37]. Knowledge on the vision capabilities of animals continues to improve despite limitations in fully understanding how they view the world because of the challenges of measuring what they perceive [38]. In fact some believe that the perception of colour vision requires some form of learning, association and consciousness [39]. Moreover, there is uncertainty as to whether animals perceive brightness and hue [39] or if colour vision is in fact important to cats and dogs [40]. Interestingly, apart from *Mustela* spp. [17] very little is known about the detection of infrared signals by animals.

In the three main species of interest to us (dogs, cats and foxes), their night visual acuity as primarily nocturnal predators is high; in the case of the cat, and more than likely foxes and dogs, their superior night vision is adapted for low visual stimuli [41]. Of most interest is the animal’s ability to detect near infrared (700–3000 nm) illumination: the part of the light spectrum used in infra-red camera traps.

### Objectives

We were interested in two critical questions related to the effect of camera trapping on predator behaviour;

1. Do camera traps produce an audible sound that animals can hear, and infrared flash illumination that they can see, and is there variability between camera trap models and modes?
2. What is the effect of the sound and illumination on animal behaviour?

To answer the first part of this question we tested a range of commonly used camera traps to determine the frequency and loudness of audio outputs and whether they fell within the hearing range of target mammals. We then tested whether the infra-red illumination from a range of models produced outputs that were within the perceptible range of known animal vision. Conducting tests on these camera traps was made possible using sophisticated technology; the challenge was obtaining enough data on vision and hearing in mammals. Our objectives were to determine whether 1) camera traps emit any sounds in the audible, infra or ultrasonic ranges for humans; 2) camera traps emit infrared illumination above the observable range of mammals; 3) mammals see or hear camera traps, 4) if there is variability in sounds and light emissions within and between camera trap models.

Two authors have suggested that human odour on camera traps may have been a deterrent to coyotes (*Canis latrans*) visiting camera trap sites [6], [9]; we constrained our investigations here to sound and light emissions. Our investigations achieved all four objectives in comprehensively reporting the sound and visual outputs of and between camera trap models, and how these outputs compare to the known hearing and visual acuity of animals.

## Materials and Methods

The main focus of this study was to evaluate the camera trap audio outputs (<20 kHz) in relationship to the known hearing ranges of animals; complementary to this was to quantify potential ultrasound outputs (20–60 kHz) and the infrared illumination spectrums for a range of camera trap models in relation to the known vision spectral data of animals.

### Camera Traps, Set-up and Triggering

We tested 12 models of camera traps for audio outputs using still and video functions; 7 models for infrared outputs and 5 models for ultra-sonic outputs (Table 1); the camera trap settings varied between models according to their specifications and functionality (see Table 1 for some details).

**Table 1:** The camera trap models and numbers used to evaluate the sound outputs in an anechoic chamber.

Make	Model	Sample	Acoustic	Infra-red	Ultrasonic	Photos/trigger	Video time	Sensitivity
Reconyx	HC600	10	•	•	•	3	NA	H
Scoutguard	550	10	•	•		3	10	H
Scoutguard	SG680 V	8*	•	•		3	10	H
Moultrie	I65	5*	•	•		1	10	H
Moultrie	I60	1		•		3	NA -	H
Cuddeback	Capture	3	•		•	1	NA	H
Picxontroller	DigitalEye	3	•			1	NA	H
Bushnell	119466	1	•	•		3	10	H
Bushnell	119456C	1	•			3	10	H
Moultrie	I40	1	•			1	10	H
Moultrie	D40	1	•	•		1	10	H
Scoutguard	560D	1	•	•	•	3	NA -	H
Uway	NT50	1	•	•	•	3	10	H
Uway	NX50	1	•			3	10	H

*Ten units of this model were tested but some failed to operate and were removed from the analysis. NA = not available.

For all measurements during the audio and infra-red optical output tests, camera traps were fixed on a tripod, 100 cm above the surface and set so that the front of the camera was 50 cm from the measuring device to optimise signal detection. Every camera was tested separately and we conducted a countdown to synchronise the measuring devices and to trigger the passive infra-red sensor (PIR), resulting in stills and/or videos being taken. To trigger the camera traps, one of the authors stood in front and to one side of the device and waved a hand across the front of the camera four times at the end of the countdown.

Every camera was tested for audio and optical outputs using still photos and where the function existed in a camera trap model, we tested video outputs.

### Acoustic Measurements

#### Auditory outputs (.01–20 kHz)

A Briel and Kjoer Type 2250 Hand held analyser was used in an anechoic chamber at the National Acoustics Laboratory in Chatswood, Sydney. The device was placed in front of the camera traps and automatically set to record camera outputs for 15 second periods. The equipment was calibrated to 94 db @1000 Hz using a Type 4230 Sound Level Calibrator.

The data were generated by the analyser using an average amplification value for each of 17 frequencies over the 15 second recording period using five measurements (L_ZFmax_, L_ZSmax_, L_ZFmin_, L_ZSmin_, L_Zeq_). Given our objective was to determine the maximum audio outputs of the cameras, we only used L_ZFmax_ values in our analysis. L_ZFmax_ is the maximum un-weighted audio level recorded over the sampling period, so it is the highest level measured irrespective of frequency.

In order to calibrate the equipment to any background sound in the anechoic chamber, we carried out ten ‘control’ recordings at each of the 17 frequencies to derive the background sound envelope, with the decibels referenced to 20 micro Pascals (20×10^−6^ Pa).

### Ultrasonic outputs (4–200 kHz)

To determine if ultrasonic frequencies were emitted by camera traps we used 10 Reconyx Hyperfire HC600, 3 Cuddeback Captures, 3 Pixcontroller DigitalEye, 1 Scoutguard 560D and 1 Uway NT50 camera traps. Control detections were also collected without a camera trap to measure any possible background sound outputs within the laboratory. As before, the camera traps were placed individually on a tripod 50 cm in front of two ANABAT Detectors connected to a ZCAIM unit. One detector was directly in front of the camera and the second at a 45 degree angle from the central axis of the camera. We tested both angles to assess whether signals were different when the devices were directly in front compared to off centre. Cameras were triggered by hand movements across the front of the camera and recordings were for 15 seconds each. Ultrasonic outputs were analysed using the acoustic analyser software, AnalookW.

### Light measurements

Tests were conducted on 32 cameras comprising 7 models (Table 1) in a laboratory at the University of New England on April 20^th^ 2011. Each camera was placed 80 cm from a hand-held ASD Field Spectrometer (FS HH 325-1075) connected to a laptop computer to enable automated data storage. Flash outputs were recorded over a 17 millisecond per acquisition period using a 10 degree field of view lens. Ten measurements per camera motion (see above) were recorded.

### Analysis

Due to the data collected by the sound analyser at 33 frequencies, we treated the audio spectrums as “functional”, thus L_ZFmax_ was a function of frequency. Analysis of the infrared illumination and ultrasonic outputs were constrained to presentation of summary statistics and raw data because the data was constrained by the unequal sample sizes of the camera trap models we had available. Furthermore, the ultrasonic data can only be reported in ANALOOK format as ranges and not as raw data.

#### Background sound

The L_ZFmax_variable was used for analysing the audio outputs in our analysis. A background sound ‘envelope’ and the 95% confidence envelope across a range of frequencies (12.5 Hz–20 kHz) was established for a set of 10 independent observations. Functional bootstrapping was applied to get the estimate of the mean curve and the associated 95% confidence curves [42].

#### Audio Outputs

Functional bootstrapping (n^boot^ = 9999) [42] was used to estimate the mean curve and confidence curves (95% CI) using the L_ZFmax_outputs for each camera trap model (stills and videos or both) where these features were available. Comparisons of still images within camera trap models were undertaken to evaluate variability using the functional mean to estimate average response for each camera trap model, and functional standard deviation to assess variability within the same models.

#### Intra-and Inter Model Comparisons

Functional *t*-tests [43] were used to compare outputs between camera traps, and between camera trap models and to the background sound envelope. Given there were 28 comparisons we predicted an increased chance of ‘false positives’, as such we adjusted the p-values using the ‘*false discovery rate*’ method [44] to account for this situation.

#### Ultrasonic Outputs

Ultrasonic camera trap outputs were recorded using an ANABAT Detector but this device does not provide raw data points and merely plots the data as a graph displaying the range of signals detected and the patterns. As such we were unable to accurately analyse variability within and between models so the data has been collated to report on the ranges detected.

#### Infrared Outputs

Summary statistics were generated for the light outputs across the range of infrared camera trap models, these are presented graphically; comparisons between infrared ranges and animal range was not undertaken in detail due to a lack of data on animal infrared vision.

#### Comparison with known animal hearing range

A mean audio output using ten HC600 Reconyx camera traps (chosen to be representative of the quietest models) was produced and compared to the published frequency hearing range of animals using a Wilcoxon test (non-parametric). We plotted mean frequency and 95% confidence intervals and used the reported hearing frequencies of 24 animals (Table 2) to determine likely relationships between hearing and sound outputs. Data available on the University of Toledo ‘Behavioural Audiograms of Mammals website (http://psychology.utoledo.edu/showpage.asp?name=mammal_hearing, accessed 6 June 2014) of known hearing ranges of animals was compared to the audio output of the camera traps. The hearing of one key species, the European Red Fox (*Vulpes vulpes*) has not been recorded in any investigations, so the known hearing range was unavailable. To overcome this constraint we extracted the calling frequencies of red foxes from published research [28], [45] using data extraction software (PlotDigitizer http://plotdigitizer.sourceforge.net, accessed 6 June 2014). We then used these data as a baseline hearing range for the red fox based on the assumption that foxes are calling to each other on this frequency and as such should hear these ranges.

**Table 2:** The approximate hearing ranges of 24 animals using data extracted from (https://www.lsu.edu/deafness/HearingRange.html) and additional data from papers cited in this study.

Animal	Scientific name	Approximate Range (Hz)	Upper Range (KHz)
bat	Unknown sp	2,000–110,000	110
cat	*Felis catus*	45–64,000	64
chicken	*Gallus gallus*	125–2,000	2
cow	*Bos taurus*	23–35,000	35
dog	*Canis lupus*	67–45,000	45
elephant	*Loxodonta sp*	16–12,000	12
ferret	*Mustela putorius furo*	16–44,000	44
guinea pig	*Cavia porcellus*	54–50,000	50
hedgehog	*Erinaceinae sp*	250–45,000	45
horse	*Equus caballus*	55–33,500	33
human	*Homo sapien*	64–23,000	23
house mouse	*Mus musculus*	2300–92,000	92
opossum	*Didelphis sp*	500–64,000	64
rabbit	*Oryctolagus cuniculus*	96–49,000	49
raccoon	*Procyon lotor*	100–40,000	40
rat	*Rattus rattus*	200–76,000	76
sheep	*Ovis aries*	100–30,000	30
cotton rat	*Sigmondon hispidusi*	1000–72,000	72
brush tailed possum	*Trichosurus vulpecula*	*??-88,000	88
fox squirrel	*Sciurus niger*	113–49,000	49
northern quoll	*Dasyurus hallucatus*	500–40,000	40
wood rat	*Neotoma floridana*	940–56,000	56
grasshopper mouse	*Onychomys leucogaster*	1850–69,000	69
kangaroo rat	*Dipodomys merriami*	50–62,000	62

*lower hearing range is unknown for this species.

## Results

We found strong evidence that animals can hear the sound of, and see the infra-red illumination of camera traps.

### Audio Outputs of Camera Traps

#### Background sounds

**Figure 1:**
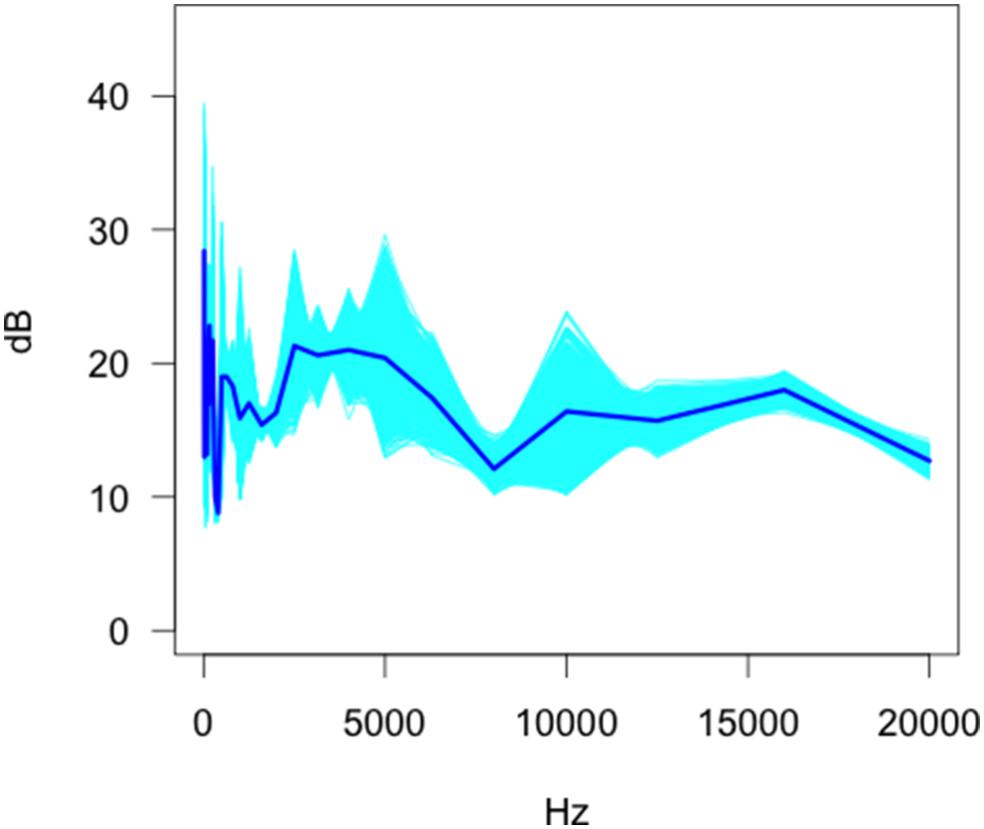
Bootstrap estimates of the functional mean of the anechoic chamber background sound envelope.

Our data show that the functional mean and standard deviation magnitudes of the background sounds were highly frequency dependent (Fig. 1). Particular frequencies tended to be associated with a higher level of average background sound (e.g. 12.5 Hz, 160 Hz, 250 Hz) with sound ranging from 8.8 dB to 28.4 dB. The variation in the sound measurements changed considerably as a function of frequency. The three largest standard deviations in sound outputs of camera traps occur at 50 Hz, 12.5 Hz, and 500 Hz, with the total range of the standard deviation estimates across frequency being 0.9–14.3 Hz. The largest mean values and the greatest standard deviation of sound components occurred at the lower frequencies suggesting that the values measured might be related to the frequency dependent accuracy of the measuring equipment.

#### Intra-camera trap comparisons - Still Images

Both the background sound and the camera models displayed means and standard deviations that were frequency dependent. Each model of camera seemed to have their own unique signature (see Table S1 and Figure S1) with characteristic peaks and oscillations. There was a semi-regular pattern to the uncertainty within a camera model, with particular sets of frequencies specific to camera model although displaying the greatest variation within camera trap models.

**Figure 2:**
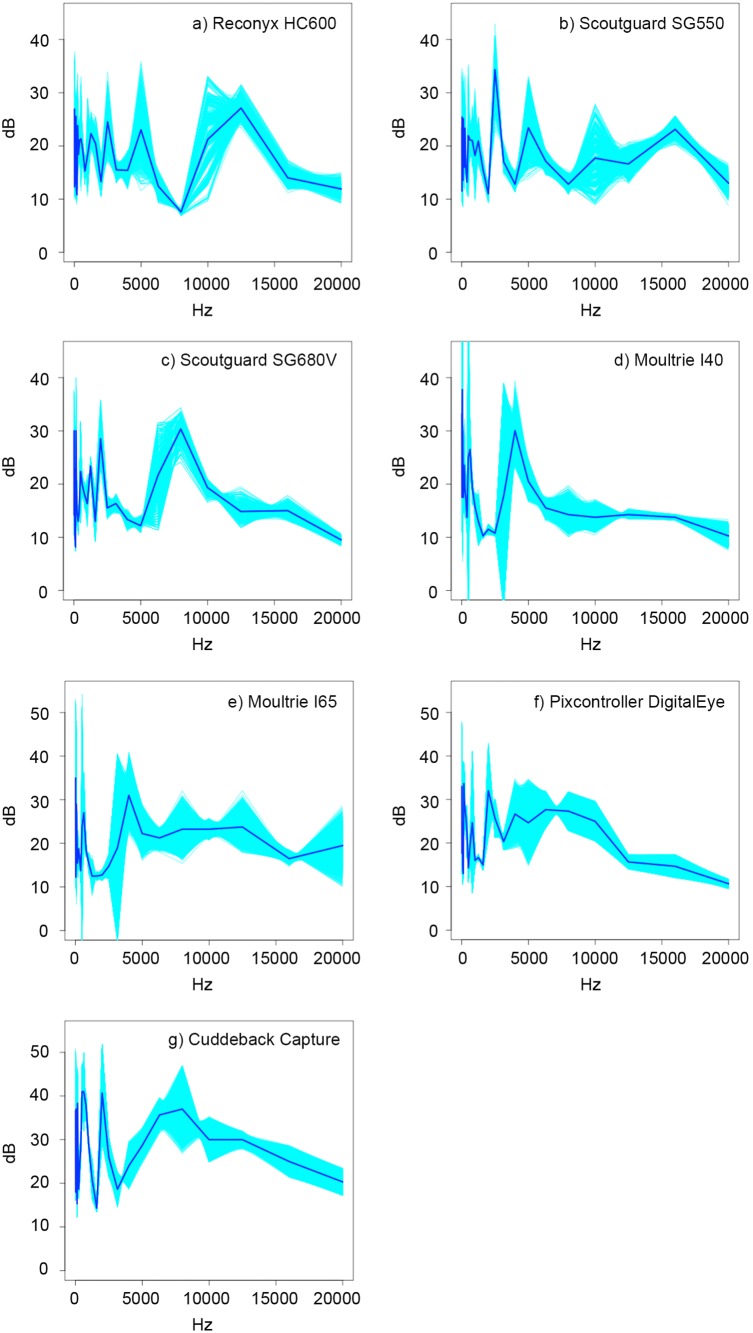
Bootstrap estimates of the functional mean (95% CI) of the sound emissions of the a) Reconyx HC600, b) Scoutguard SG 550, c) Scoutguard SG680 V, d) Moultrie I40, c) Moultrie I65, e) Pixcontroller DigitalEye and f) Cuddeback Capture taking still photos.

In analysing 10 Reconyx HC600 we established that the mean values range from 7.6 dB to 27.1 dB across the frequency range (12.5 to 20,000 Hz) with a standard deviation ranging from 0.8 dB to 17.2 dB (Fig. 2). There was a substantial difference in the magnitude of the camera output with the top three ‘loudest’ frequencies (12,500 Hz, 12.5 Hz, and 25 Hz) and the three highest standard deviation values 10,000 Hz, 5,000 Hz, and 25 Hz.

The Scoutguard SG 550 (still) functional mean was frequency dependent with spikes at 2500 Hz, 5000 Hz, and 10,000 Hz, although different to the HC600 (stills) and the background sound (Fig. 2). The functional standard deviation was also frequency dependent and similar to the HC600. At 250, 5000 and 10000 Hz the variability in the output (L_ZFmax_) between cameras was greatest within a model (i.e., Reconyx still, Scoutguard still). The range of the functional mean was from 11.0 dB to 34.3 dB, with the top three highest values occurring at 2,500 Hz, 31.5 Hz, and 125 Hz.

The Scoutguard KG680 V had a unique ‘signature’ as well as frequency dependent characteristics in the functional mean. The range of the functional mean was 8.1 dB to 30.2 dB with the three highest values occurring at 8,000 Hz, 160 Hz, and 12.5 Hz. These frequencies show the greatest variability within a model and are quite different to the HC600 and SG 550. The functional means of the Moultrie I40 display frequency dependency. The signature was also unique but due to the very small sample size (n = 4) there is some uncertainty in the estimates.

We found that there were sharp and sudden shifts in the L_ZFmax_statistic for different frequencies in this model. The range of the functional mean was 10.3 dB to 37.8 dB whilst the functional standard deviation ranged from 0.4 dB to 16.3 dB. The three greatest values of the functional mean occurred at 50 Hz, 40 Hz, and 12.5 Hz. The greatest variability occurred at 3150 Hz, 500 Hz, and 80 Hz in this order. Similarly, there was a frequency dependence and unique sound signature in the Moultrie I65 camera trap The range of the functional mean was from 12.3 dB–35.0 dB with the top three values occurring at 12.5 Hz, 4000 Hz, and 80 Hz.

The Pixcontroller DigitalEye also had a unique sound signature and shared some similarities with the background sound profile. It also exhibited frequency dependent structure in both the functional mean (10.7 dB–33.7 dB, maximal values at 160 Hz, 12.5 Hz, and 2000 Hz.) and standard deviation (0.5 dB–10.3 dB) with the top three values occurring at 800 Hz, 63 Hz, and 12.5 Hz.

Three Cuddeback cameras produced functional mean ranges from 14.3 dB to 41 dB with the top three values occurring at 400 Hz, 500 Hz (equal highest), and 2000 Hz (functional standard deviation 0.5 dB–7.9 dB with the three largest values occurring at 12.5 Hz, 2000 Hz, and 8000 Hz.

### Comparisons between camera traps in still modes and background sounds

**Figure 3:**
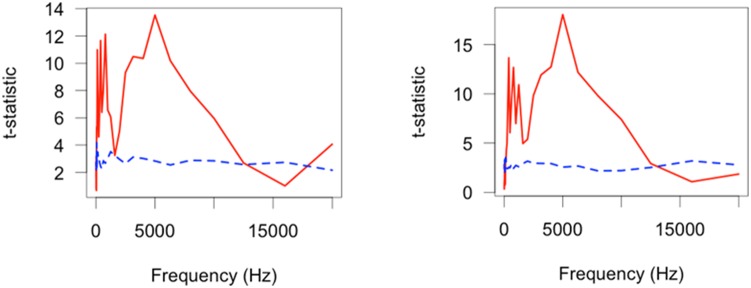
Functional t-test results as a function of frequency for two select contrasts: a) Background sound- Cuddeback and b) HC600-Cuddeback. The red (solid) line indicates the permutation statistic (t_max_) results for 999 random permutations of the input data sequence whilst the blue (dashed) line indicates the α = 0.05 critical level as a function of frequency. When the red (solid) line is equal or above the blue (dashed) line there was a significant difference at that frequency.

There were several contrasts displaying significant differences between models (Table 3). Specifically, comparisons between the Background-Cuddeback, HC600-MI40, HC600-Cuddeback, SG550-Cuddeback, KG680 V-Cuddeback, MI65-Cuddeback, and Pixcontroller-Cuddeback were statistically significantly different. There were a further seven contrasts that seem to be statistically significant different but they did not pass the multiple comparisons adjustment. There was a significant difference in the background sound and the Cuddeback, which consistently produced louder sound outputs (16 Hz: Background – 15.9 dB Cuddeback- 23.7 dB, 80 Hz: Background- 14.9 dB Cuddeback- 29.0 dB, 400 Hz: Background – 8.8 dB, Cuddeback- 28.0 dB) (Fig. 3). The contrast between Reconyx HC600- Moultrie MI40 could either be a statistical anomaly or could indicate a difference in the operational frequency response for these two cameras, which is so minute that it is within the variation of the background sound envelope. These analyses confirm that different camera models exhibit unique sound profiles but not discernibly different to the variability within models.

**Table 3:** Comparisons between different camera outputs (still) and the background sound envelope.

Model 1	Model 2	Statistic	p-value	Adjusted p-value
Background	HC600	t_max_ = 5.43	<0.01*	0.056
Background	SG550	t_max_ = 2.73	0.22	1.00
Background	KG680 V	t_max_ = 4.62	0.02*	0.53
Background	MI40	t_max_ = 5.14	0.02*	0.62
Background	MI65	t_max_ = 4.47	0.06	1.00
Background	Pixcontroller	t_max_ = 4.27	0.13	1.00
Background	Cuddeback	t_max_ = 13.53	<0.01*	0.00 *
HC600	SG550	t_max_ = 3.44	0.07	1.00
HC600	KG680 V	t_max_ = 2.99	0.20	1.00
HC600	MI40	t_max_ = 8.70	<0.01*	0.00*
HC600	MI65	t_max_ = 3.60	0.17	1.00
HC600	Pixcontroller	t_max_ = 5.89	0.03*	0.76
HC600	Cuddeback	t_max_ = 18.03	<0.01*	0.00*
SG550	KG680 V	t_max_ = 3.10	0.17	1.00
SG550	MI40	t_max_ = 6.74	0.01*	0.17
SG550	MI65	t_max_ = 3.561835	0.16	1.00
SG550	Pixcontroller	t_max_ = 5.09	0.11	1.00
SG550	Cuddeback	t_max_ = 15.10	<0.01*	0.00*
KG680 V	MI40	t_max_ = 2.40	0.56	1.00
KG680 V	MI65	t_max_ = 3.53	0.17	1.00
KG680 V	Pixcontroller	t_max_ = 5.01	0.04*	1.00
KG680 V	Cuddeback	t_max_ = 15.10	<0.01*	0.00*
MI40	MI65	t_max_ = 3.81	0.12	1.00
MI40	Pixcontroller	t_max_ = 4.16	0.21	1.00
MI40	Cuddeback	t_max_ = 11.00	0.03*	0.84
MI65	Pixcontroller	t_max_ = 4.38	0.08	1.00
MI65	Cuddeback	t_max_ = 12.99	<0.01*	0.00*
Pixcontroller	Cuddeback	t_max_ = 21.97	<0.01*	0.00*

Test statistics (t_max_) falling below the critical value are not significant at the particular frequency whilst those at or above were considered significant. (*denotes significance at p<0.05).

We found that for most frequencies, particularly the low to medium frequencies, significant differences exist.

### Intra camera trap comparisons - Video

**Figure 4:**
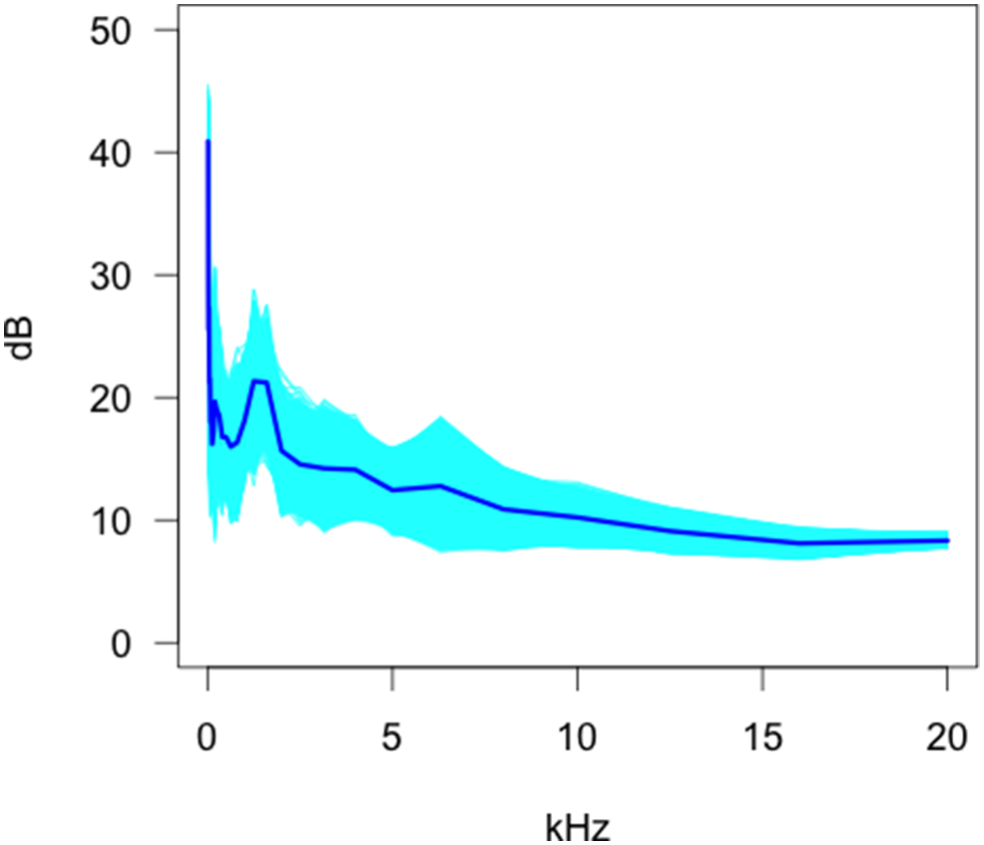
Bootstrap estimates of the functional mean and 95% confidence envelopes for the Scoutguard SG550.

Of the four camera trap models tested, the Scoutguard SG550 (Fig. 4) showed an overall decreasing trend with an occasional minor peak where the operational sound or the uncertainty in the estimate or uncertainty between models was higher. The operational sound characteristics appeared similar to that of the Moultrie MI40 but the functional standard deviation was higher at lower frequencies.

**Figure 5:**
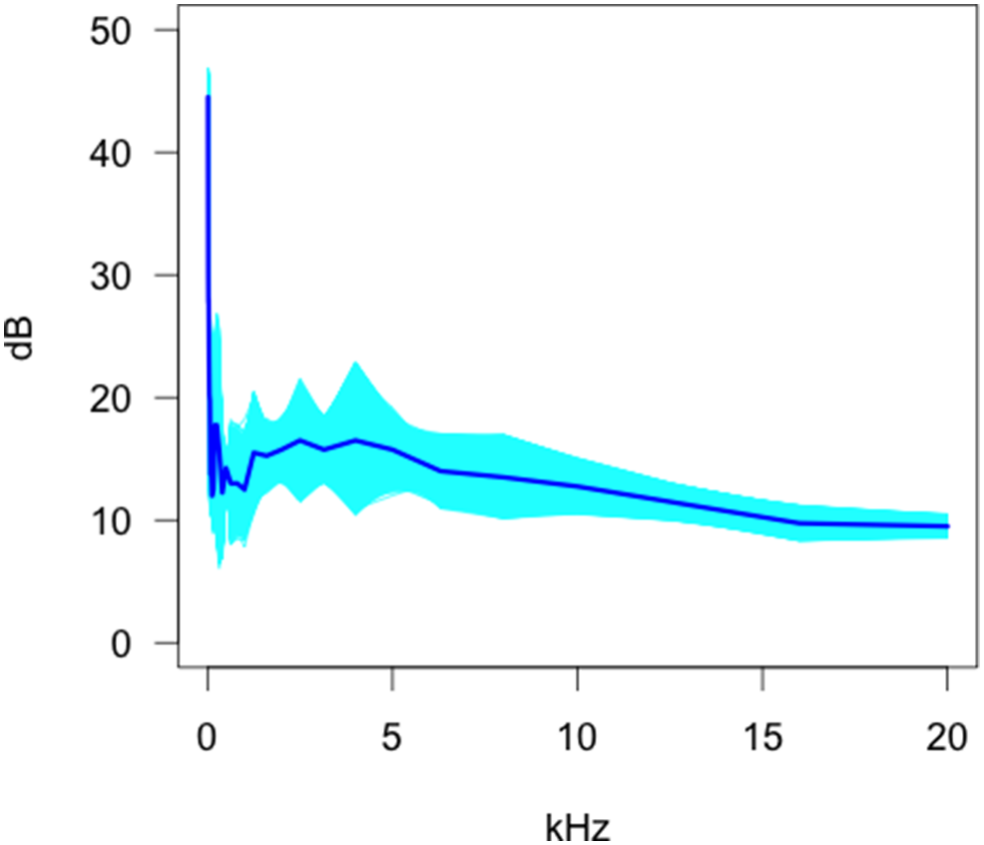
Bootstrap estimates of the functional mean and 95% confidence envelopes for the Scoutguard KG680.

In the Scoutguard KG680, the functional mean exhibited a slow decrease in operational sound level with frequency (Fig. 5). Of note was the relative tight envelope around the estimate of the mean indicating that this curve was estimated with far less uncertainty than the other models.

**Figure 6:**
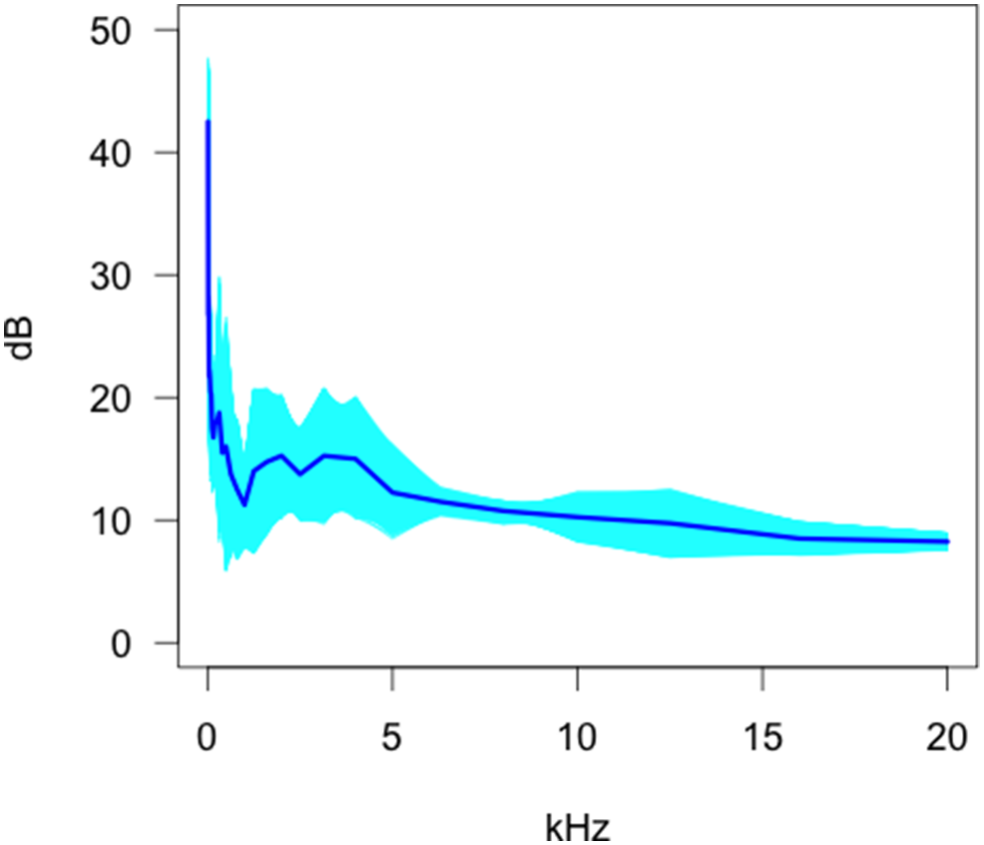
Bootstrap estimates of the functional mean and 95% confidence envelopes for the Moultrie MI40 camera.

In the Moultrie MI40 the functional mean of the sound is highly frequency dependent (Figures 6 and Table 4). The operational sound of the camera and the variation between models vary with frequency. The highest operational sound occurred at the lowest frequencies as well as the greatest variation and uncertainty between models.

**Table 4:** Comparisons between different video camera outputs as well as the background (*: denotes statistical significance below the p = 0.05 level).

Model 1	Model 2	Statistic	p-value	Adjusted p-value
Background	MI40	t_max_ = 4.15	0.08	0.75
Background	MI65	t_max_ = 4.89	0.03*	0.28
Background	SG550	t_max_ = 3.02	0.17	1.00
Background	KG680 V	t_max_ = 2.88	0.41	1.00
MI40	MI65	t_max_ = 4.33	0.13	1.00
MI40	SG550	t_max_ = 10.24	<0.01*	0.02*
MI40	KG680 V	t_max_ = 3.71	0.21	1.00
MI65	SG550	t_max_ = 4.32	0.05	0.52
MI65	KG680 V	t_max_ = 3.71	0.12	1.00
SG550	KG680 V	t_max_ = 3.50	0.21	1.00

**Figure 7:**
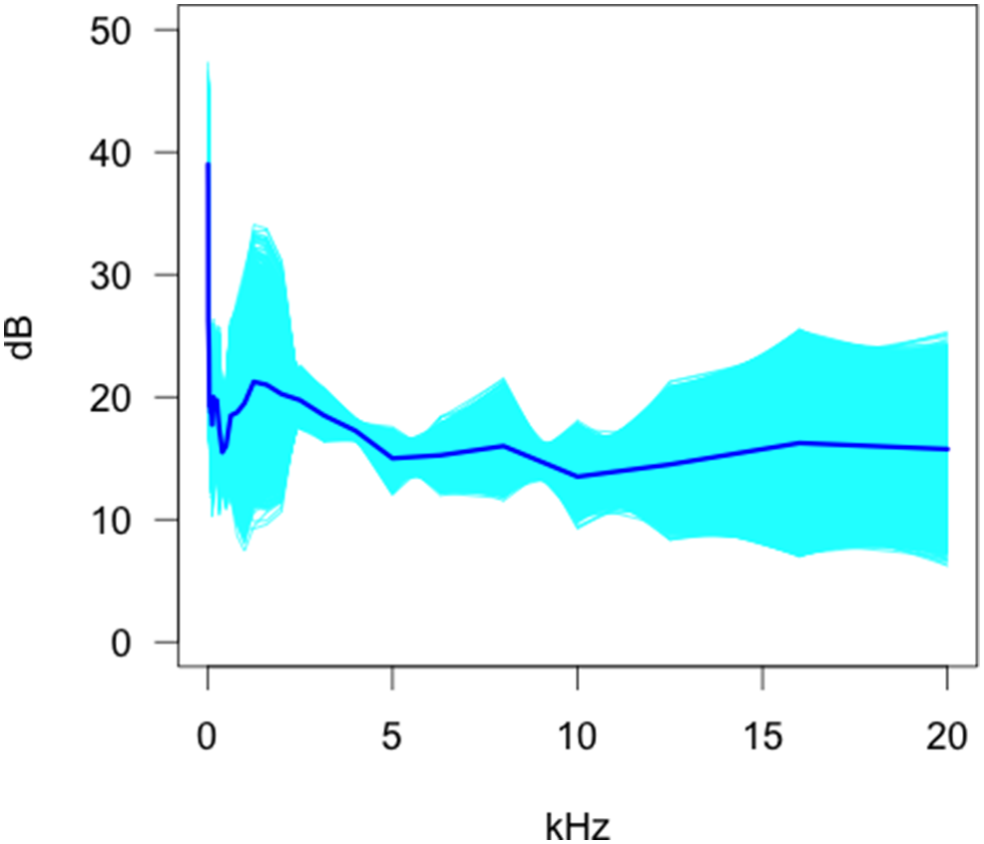
Bootstrap estimates of the functional mean and 95% confidence envelopes for the Moultrie MI65 camera.

A slight frequency dependent response was observed in the Moultrie MI65 (Fig. 7) around the mean, but the standard deviation estimate was highly frequency dependent with a pronounced peak occurring in the 0–3 kHz band and a general linear increase occurring from 4–20 kHz. The increasing width of the 95% confidence envelope as frequency increases reflects uncertainty with increasing frequency. This might be due to low sample sizes and our inability to establish whether or not the mean curve increases, remains stationary, or decreases at these frequencies. These data show that the sound level for the MI65 is higher than the MI40.

Estimates of the mean audio output and standard deviation were estimated as a function of frequency for four different camera models operating in video mode. All cameras appear to have frequency dependent operational characteristics and furthermore there appears to be differences in the mean sound levels between models. Importantly, the standard deviation curve estimates within a model appear greater or on similar magnitude to the mean differences between cameras. This could be due to the limited number of camera traps models, although this is unlikely because the analysis suggested wide variation in magnitude and form.

### Comparisons between Video Modes and Background Sound

**Figure 8:**
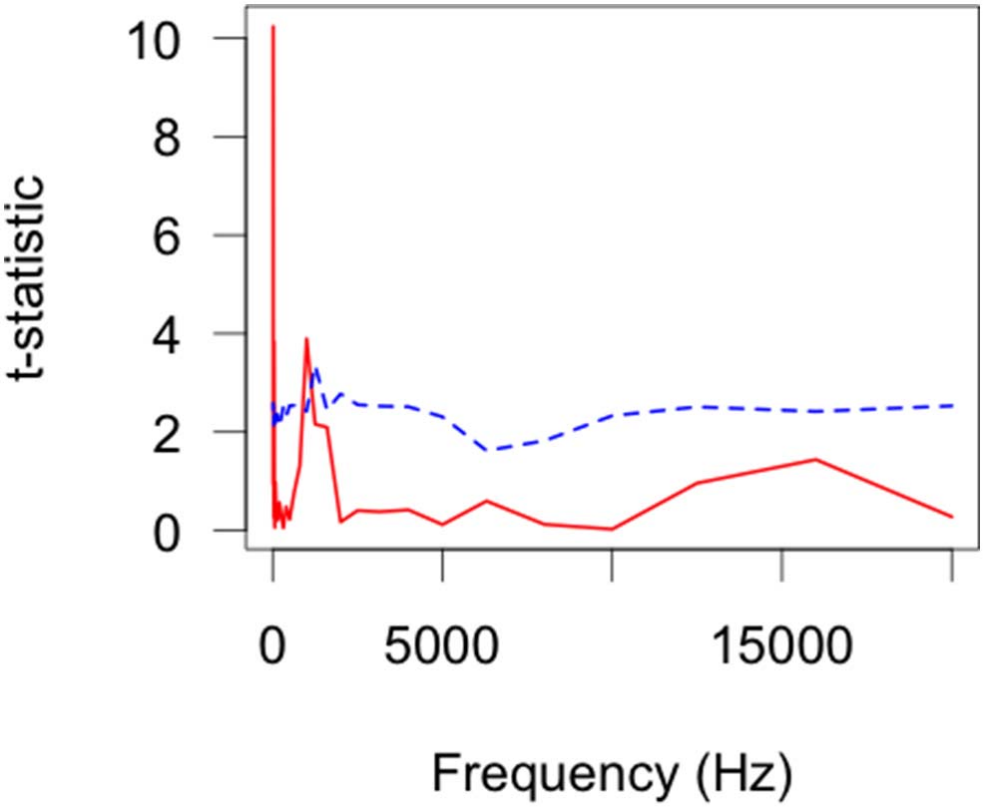
Functional t-statistics as a function of frequency for the MI40-SG550 V contrast in video mode. The test statistic (t_max_) is displayed as a solid line and the α  = 0.05 critical value as a function of frequency is displayed as a dashed line.

Our functional tests with multiple comparison corrections for background sound in video mode showed no significant difference (Table 4). There was however a difference in audio outputs between the MI40 and SG550 with a difference in the response around 1000 Hz but it was still within the range of the background sound (Fig. 8).

Except at very low frequencies and a slight (t_max_≈1–1.5) variation around 1000 Hz, there were no significant differences in response across frequencies. The secondary peak at 1000 Hz is indicative of a difference in the functional mean estimates for these two cameras in video mode.

### Comparisons between Still and Video Modes

There was no difference in still and video frequency response or between the four camera models (Scoutguard and Moultrie) (Table 5).

**Table 5:** Still vs Video comparisons using functional t-tests (*: denotes statistical significance below the p = 0.05 level).

Model	Statistic	p-value	Adjusted p-value
MI40	t_max_ = 2.17	0.74	1.0
MI65	t_max_ = 2.99	0.41	1.0
SG550	t_max_ = 1.95	0.73	1.0
KG680 V	t_max_ = 2.98	0.35	1.0

### Ultrasonic recordings

Ultrasound frequencies tests on the five camera trap models confirm that camera traps do produce ultrasonic outputs each time a photo is taken (Table 6). Frequency ranges for Reconyx HC600 was 3–60 kHz with a median output of 52.5 kHz (SD = 13.4) directly in front of the device and 47.5 kHz (SD = 7.3) perpendicular to the device. Other models emitted outputs within a similar range. There was some variability within models due to the method of measuring the outputs; ANABAT detectors are designed to measure bat echolocation not low level ultrasonic sound.

**Table 6:** Ultrasonic outputs from five camera trap models including two control recordings, two ANABAT directions were utilised (directly in front and offset 45 degrees to the central axis of the camera).

	directly in front		off-set 45 degrees	
Model and Code	Lower (kHz)	Upper (kHz)	Lower (kHz)	Upper (kHz)
HC600–1	3	35	3	40
HC600–2	3	20	3	35
HC600–3	3	50	3	45
HC600–4	3	55	3	50
HC600–5	3	60	3	55
HC600–6	3	55	3	0
HC600–7	0	0	0	0
HC600–8	3	55	3	50
HC600–9	0	0	0	0
HC600–10	3	50	3	45
Cuddeback-1	3	40	3	55
Cuddeback-2	3	40	3	60
Cuddeback-3	3	60	3	40
Pixcontroller-1	0	0	0	0
Pixcontroller-2	0	0	3	45
Pixcontroller-3	3	35	0	0
Scoutguard 560D-1	3	0	0	0
Uway NT50–1	3	35	3	0
Control 1	0	0	0	0
Control 2	0	0	0	0

### Audio Outputs and Known Hearing Ranges of Animals

**Figure 9:**
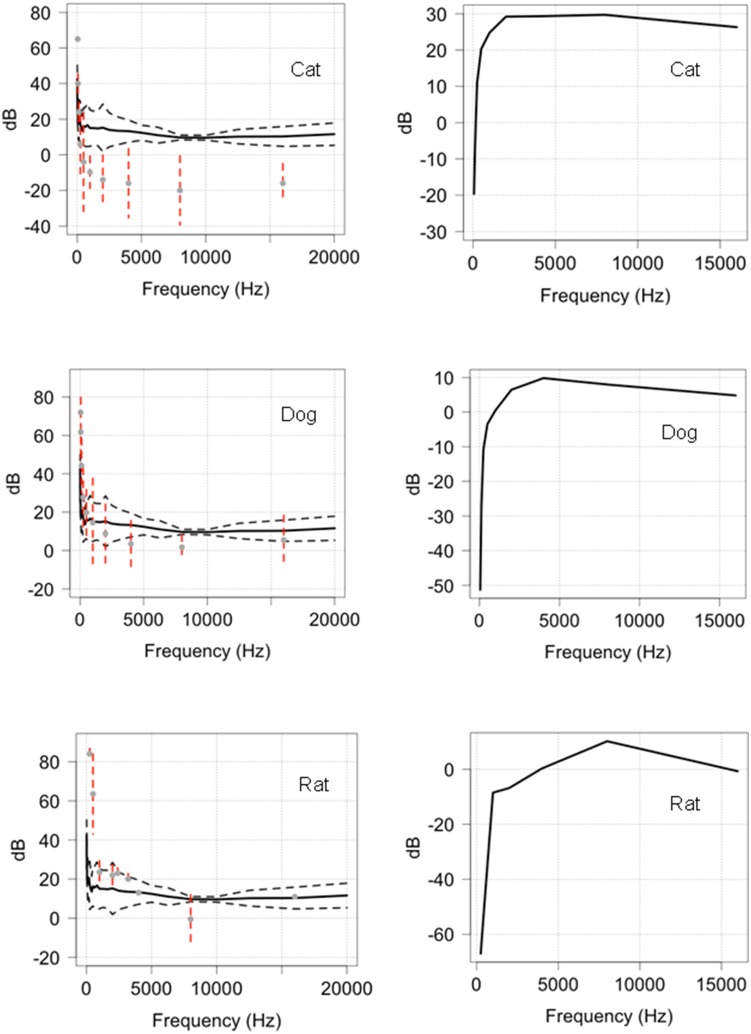
Dog (1), cat (2) and rat (3) hearing ranges in relation to the outputs of HC600 camera traps (1a, 2a, 3a) and as a function of frequency (1b, 2b, 2c). The black line is the mean audio output of the camera trap; the grey dotted lines are the 95% confidence limits. The red dotted lines represent the standard error around the known hearing range of the dog, cat and rat. Where the grey points and red dotted lines (SE) are below and closest to the mean audio output of the camera, the sound can be detected by the animal.

**Figure 10:**
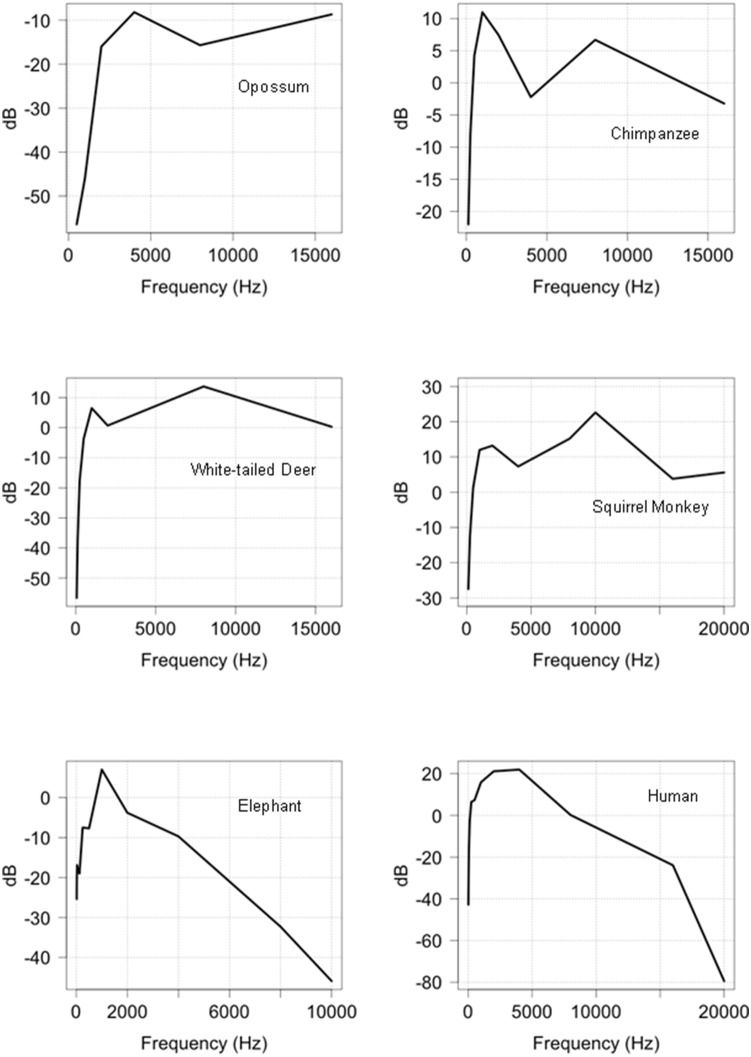
The auditory threshold of 6 mammals represented as a function of frequency.

**Figure 11:**
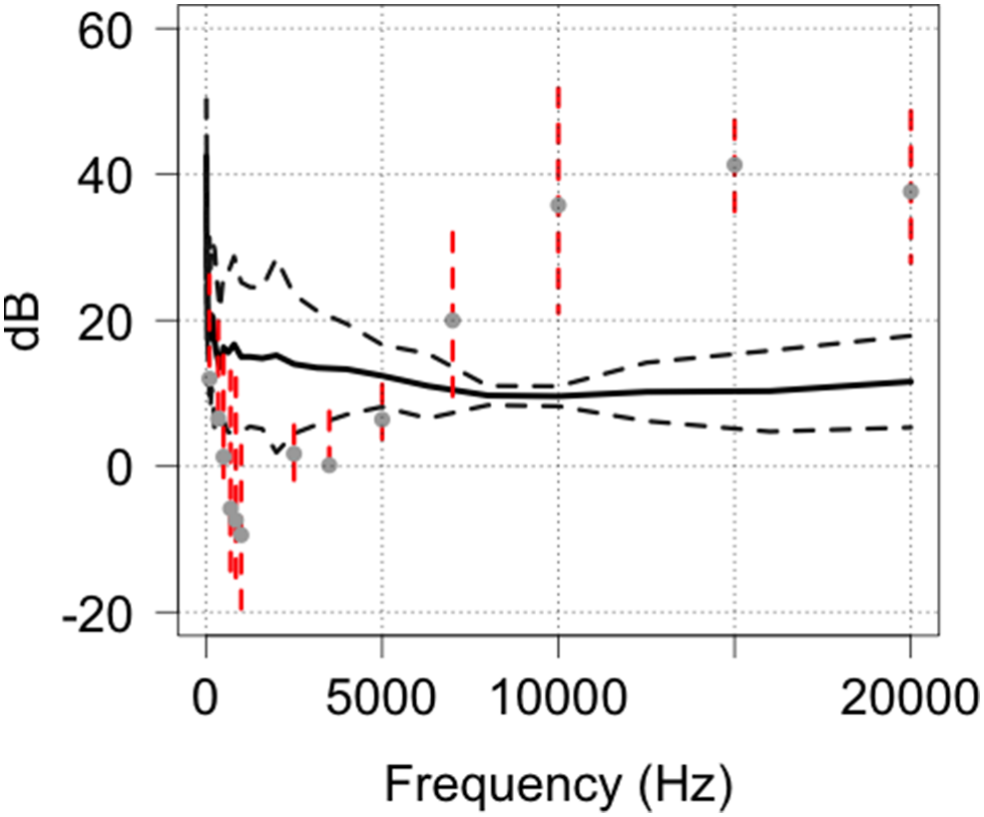
Comparison of the predicted hearing range of the red fox in relation to the outputs of HC600 camera traps and as a function of frequency.

Our tests comparing Reconyx (HC600) camera trap outputs to the existing hearing ranges of 21 species (see http://psychology.utoledo.edu/showpage.asp?name=mammal_hearing, accessed 6 June 2014) found compelling evidence that camera trap sound outputs fall within the hearing range of most of the species (Fig. 9–11). In 9b, 9b and 9c we have presented data to show the relationship between the camera sound and the auditory threshold of the animal as a function of frequency. These data strongly suggest that dogs, cats and rats have the capacity to detect low frequency outputs (<20 kHz) from camera traps. Data presented in Figure 10 provide evidence that a further six mammals, including humans, have the capacity to detect camera traps in the lower frequency bandwidths. The hearing ranges in comparison to camera trap outputs for a further 12 mammals are provided in Figure S2.

Given the paucity of data on the known hearing range of red foxes we carried out a comparison on the minimal hearing range for this species based on their calling frequencies [25], [45] (Fig. 11). This was based on the assumption that foxes must be able to hear the frequency of fox calls recorded at a minimum. From this analysis we derived an optimum hearing range for the red fox of around 8–12 kHz although this would be an under-estimation of their true range. Despite having to use call frequencies to model hearing range, the results show that red foxes can easily hear camera trap outputs.

The data presented provide robust evidence that mammals can detect the sound outputs of camera traps.

### Infra-red Wavelength Outputs

**Figure 12:**
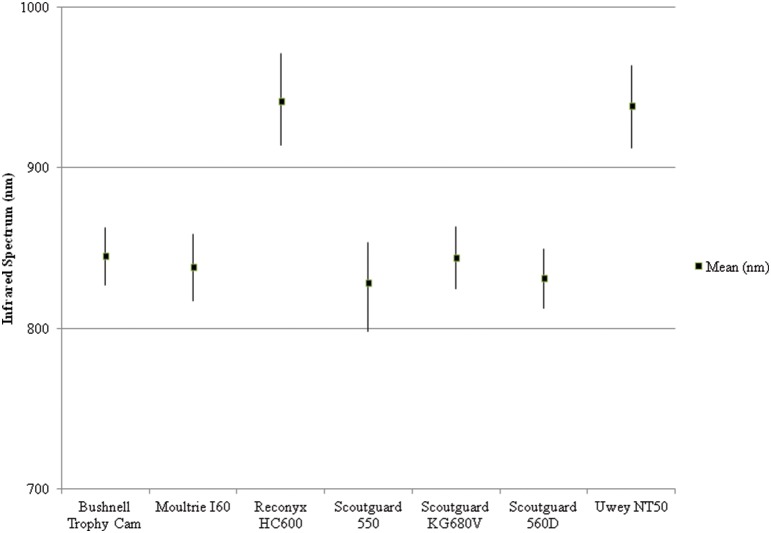
Mean infra-red wavelength illumination (nm) outputs for seven camera trap models showing the highest and lowest values.

The infra-red illumination ranges varied between models (Fig. 12) but there was no difference in wavelength outputs within models for the Reconyx HC600 (Mean = 940.5, SD = 1.8, 95% CI = 1.3), Scoutguard 550 (Mean = 828.3, SD = 4.7, 95% CI = 3.4) and Scoutguard 680 V (mean = 844.1, SD = 0.6, 95% CI = 0.5) camera traps.

Based on the data in Figure 12, camera traps that are advertised as “no glow” (HC600) or “black ops” (NT50) are clearly using infrared technology with wavelengths operating above 850 nm. These infrared LED’s are emitting light that is nearly invisible to the human eye, but not some animals.

## Discussion

In this study we tackled the first part of a two staged question; do camera traps have the capacity to project audio and optical stimuli to wildlife? Moreover, are these outputs different between and within models and recording modes (still or video)? We present these data on the audio and visual outputs of camera traps to highlight the importance of identifying the effects camera trapping may have on animal behaviour.

### Animal Hearing

A wide range of comparisons were conducted to investigate the possibility of differences in operating audio outputs of different camera models in both still and video mode. In the vast majority of cases (except the Cuddeback) the operational sound was little different from the background sound in the sound laboratory. In some cases slight differences were found between models (e.g. MI40 and SG550 video) but were of such a low level that they were within the magnitude of the background sound. The noise created by two people being present in the anechoic chamber conducting the experiments probably produced sounds and affected the background sound envelope. If we were able to conduct the tests remotely there may have been a more significant difference between camera trap noise and background noise.

In comparing the auditory range of animals in contrast to our recorded outputs, we sourced sonagraph data for a range of species. There have not been any sonagraphs to determine the hearing ranges of foxes although it has been reported that red fox have an upper limit of 65 kHz [37]. While studies of 75 foxes [46] reported the frequency of a range of calls made by foxes to be less than 2 kHz, which is consistent with one other study [45] that reported calls all under 2.5 kHz. The foxes hearing capacity has been reported to have a reduced capacity between 5–11 kHz with a discernible reduction around 8.5 kHz, but reported that they hear sounds well at 10–14 kHz [25]. Given red fox calling frequencies, they would certainly hear some of the infra and ultra sounds emitted by camera traps. We report the first evidence that animals can detect the presence of camera traps due to the audio and optical outputs from these devices. This study determined that at certain frequencies, animal hearing (Table 2 and see http://www.lsu.edu/deafness/HearingRange.html, accessed 6 June 2014) can easily detect these sounds.

The results of our testing also provide conclusive evidence that camera traps do emit ultrasonic outputs, especially when battery levels are low. In a pilot trial we found that low powered batteries resulted in the ANABAT detecting an output but in subsequent tests with fully charged batteries, there was no audible signal suggesting that camera trap outputs vary with battery life. The use of a bat call monitoring device has been used previously to test LED lights being used in research on *Mustella* vision [17]. The authors were unable to detect any outputs by the lights, however in the case of camera traps there are a range of electrical and mechanical components apart from the LED circuitry that may be emitting sound.

### Animal Vision

Information on the extent of infrared detection by other mammals is scant in the literature. There have been some investigations using behavioural methods that report some animals can see infrared light in the range 539–870 nm although the evidence is limited across the taxa. As such we were unable to conduct any comparative analysis of infrared flash light outputs with animal vision to test our hypotheses.

Research has established that Honey Possums (*Tarsipes rostrata*) are able to see light in the 557 nm range [18] while ferrets (*Mustela furo*) can see around 870 nm [17] and Tamar Wallaby (*Macropus eugenii*) peaked at 539 nm [20].

**Figure 13:**
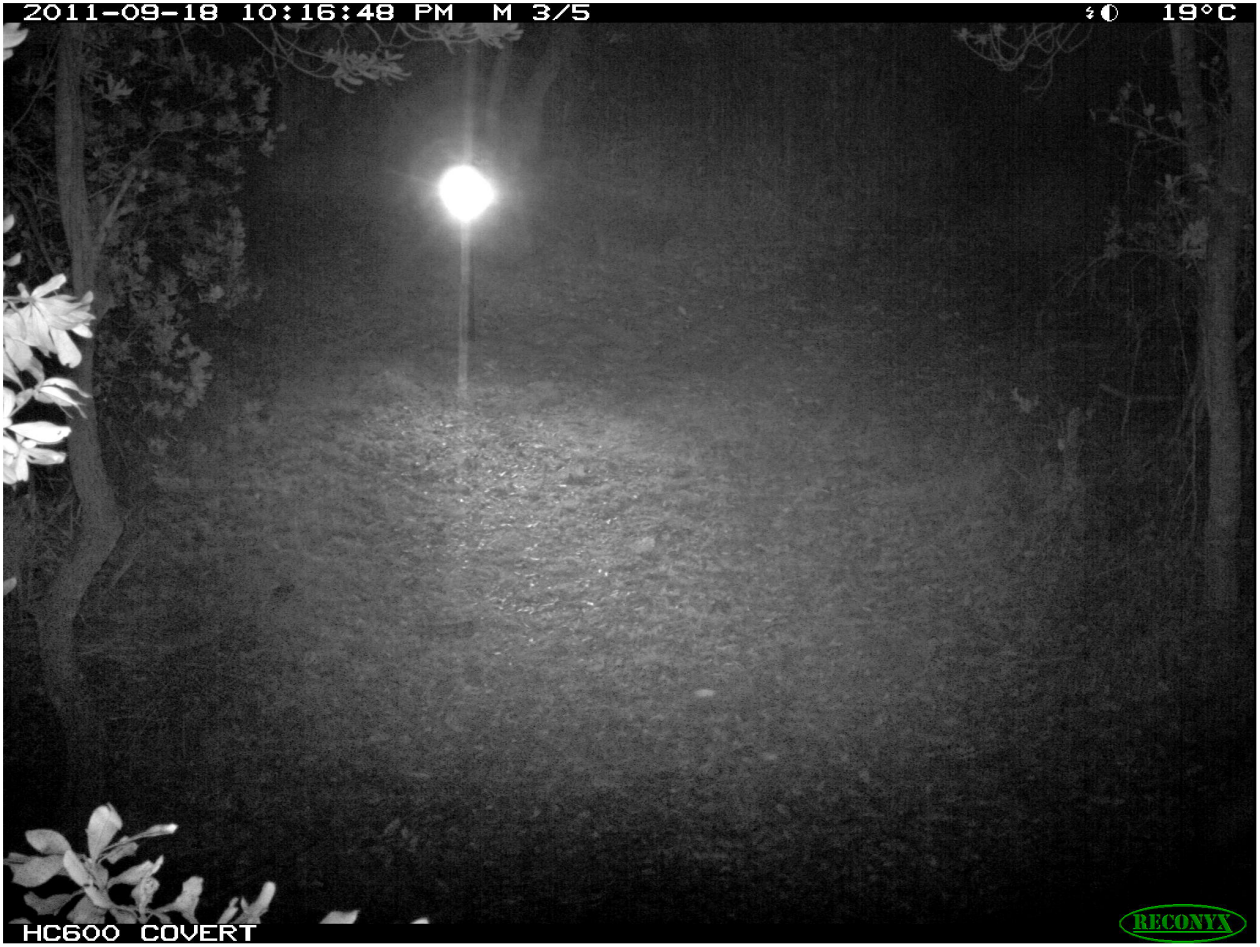
Infra red illumination of two opposing Reconyx HC600 camera traps simultaneously triggering.

These data probably underestimate the extent of an animal’s ability to detect infrared light since they are based on behavioural studies [38], not physiological analysis, because such technology is unavailable. As such, we are unable to state exactly what the limits of animal vision might be, and we believe that the range of infrared light presented in Figure 12 are likely seen by many species of animal. In support of this claim, one of the authors (PM) was able to see a faint red glow of a Reconyx HC600 in absolute darkness. Reports of humans detecting infrared (1064 nm) well above the illumination currently used in camera traps have been recorded [47], [48]. This being the case, there is no doubt that nocturnal animals with vision sensitive to night light can see infrared illumination. The responses of animals to infrared flash are highly variable between species and individuals (Meek Unpub data; Ballard Unpub data). While we cannot measure exactly what animals see, they most likely see a similar image to the flash recorded by two camera traps triggered simultaneously, as shown in Figure 13.

Cats appear to detect the presence of camera traps more than other animals (Meek Unpub data; Ballard Unpub data), which is probably due to their retina sensitivity at 826 nm [49] and total vision field of view being 287° with binocular over lap of 130° [37]. This peripheral view combined with the very high sensitivity to infrared light at the higher end of the near infrared spectrum would make cats more than capable of easily detecting camera trap flashes; especially in models with light emissions below 800 nm (see Fig. 12).

The effects of white flash camera traps on animal behaviour have been recognised as an intrusive survey method because it has been shown to startle and cause animals to flee (7). Some authors have suggested that using infrared illumination may reduce this flight response [7], [8], [14], especially where infrared wavelengths exceed ∼870 nm [17]. While there is little information on the detectable range of infrared wavelengths by most animals, one study did find that ferrets’ (*Mustelo furo*) maximum observable range was about 870 nm [17]. Multiple images and corresponding footprint detecting plots from our research on feral cats, wild dogs and foxes in Australia over several years indicates that all three species can detect flash illumination from Reconyx HC600 camera traps (Meek Unpub data; Ballard Unpub data). In field trials where two HC600 were facing each other, we were able to accidentally trigger the cameras to simultaneously trigger showing visually what nocturnal animals may see when infra red illumination occurs (Fig. 13) [50].

Anecdotal reports of ship rats (*Rattus rattus*) and brush-tailed possums (*Trichosurus vulpecula*) from three unpublished studies describe avoidance of infrared illumination in these species (see [17]). Although there has not been any effect found on predator behaviour around ground-nesting bird nests from infrared camera traps used to detect visitation [51].

Despite wide spread belief that humans cannot see near infrared light, many authors have reported being able to detect infrared light during experiments and these descriptions have been described [17]. On the evidence presented in the literature and summarised here, we conclude that most nocturnal or arrhythmic (nocturnal with some diurnal activity) mammals can see the infrared illumination (flash) emitted by camera traps.

### Conclusions

Hearing and vision work together to form what is referred to as auditory localisation acuity [22]; where an animal hears a sound and turns towards the sound using eye sight to focus in on the stimuli. This is probably the case in camera trapping, where a sound is heard by a passing animal and the device is further recognised by vision, thus enabling animals to detect the device.

With the constant sounds of the forest animals are unlikely to be hearing the camera traps constantly as the frequency and amplitude values are very similar. Furthermore, the audio outputs collected in the anechoic chamber were recorded at 50 cm, and it is reported that with every metre away from the camera a loss of 6 dB is expected [52]. Sound levels are affected by distance from the source, atmospheric attenuation, terrain, ground cover, wind and weather [53], forest density (a function of limb and trunk density) and foliage [54] and as such we acknowledge that this attenuation may reduce sounds from camera traps under field conditions. This is because unlike the pure sounds recorded by audiograms, complex sounds like those in a natural setting where multiple frequencies and background scatter exist are less likely to be detected by animals [55].

In some studies the target species’ ability to detect a camera trap may not be important because the requirement is to detect presence only, so irrespective of whether the animal baulks and runs from a camera trap is of no importance. Where repeat visits to a site are imperative for analysis, i.e., mark recapture, photographic indexes, CPUE and activity indexes, the interference to behaviour and potential avoidance of the camera trap may introduce a bias on the probability of detection. An issue also raised by in one study [17] in regard to the potential for infrared light emitting surveillance devices or traps to cause avoidance by animals.

There is a convincing argument presented in this study to confirm that most mammals can hear the operational sounds generated by camera traps in both the infrasound and ultrasound ranges. Moreover, given the strong relationship between vision and hearing acuity [22], this study concludes that most mammals can see the infra-red illumination used in camera traps.

## Supporting Information

Figure S1 **The noise frequency outputs of twelve camera trap models and the background control.**
(TIF)

Figure S2 **The hearing range of an additional twelve animals in comparison to the noise outputs of a camera trap.**
(TIF)

Table S1 **The mean audio outputs of 12 camera trap models at different frequencies.**
(DOCX)
