# Supplementary figures and images for: Camera Traps Can Be Heard and Seen by Animals
Source: PLoS One. 2014 Oct 29;9(10):e110832. doi: 10.1371/journal.pone.0110832 (PMC4212972; doi:10.1371/journal.pone.0110832)

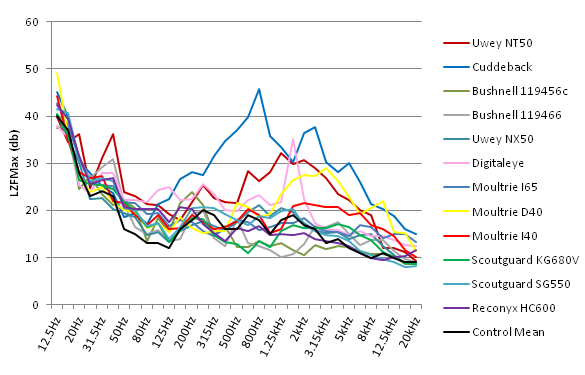

Supplement: Figure S1 — The noise frequency outputs of twelve camera trap models and the background control. (TIF) [file pone.0110832.s001.tif]

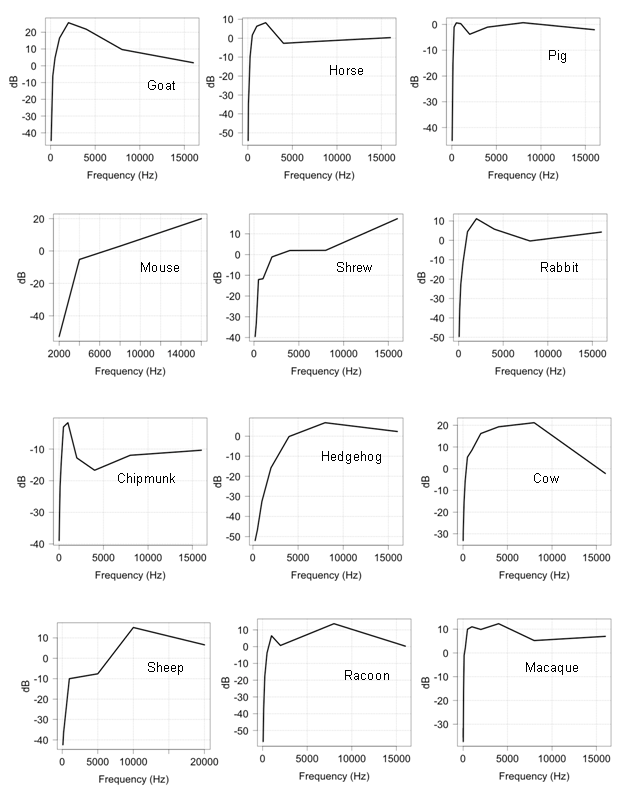

Supplement: Figure S2 — The hearing range of an additional twelve animals in comparison to the noise outputs of a camera trap. (TIF) [file pone.0110832.s002.tif]
