# Supplementary material for: Camera Traps Can Be Heard and Seen by Animals
Source: PLoS One. 2014 Oct 29;9(10):e110832. doi: 10.1371/journal.pone.0110832 (PMC4212972; doi:10.1371/journal.pone.0110832)
Supplement: Table S1 — The mean audio outputs of 12 camera trap models at different frequencies. (DOCX) [file pone.0110832.s003.docx]

**Appendix 1.** The mean audio outputs of 12 camera trap models at different frequencies.

| Frequency | Uwey NT50 | Cuddeback  Capture | Bushnell 119456c | Bushnell 119466 | Uwey NX50 | Digital-eye | Moultrie I65 | Moultrie D40 | Moultrie I40 | Scoutguard KG680V | Scoutguard SG550 | Reconyx HC600 | Control Mean |
| --- | --- | --- | --- | --- | --- | --- | --- | --- | --- | --- | --- | --- | --- |
| 12.5Hz | 40 | 45 | 37 | 40 | 40 | 38 | 43 | 49 | 44 | 41 | 42 | 42 | 40 |
| 16Hz | 34 | 40 | 38 | 39 | 35 | 36 | 38 | 36 | 35 | 36 | 41 | 39 | 37 |
| 20Hz | 36 | 30 | 25 | 31 | 30 | 25 | 31 | 27 | 28 | 26 | 31 | 31 | 28 |
| 25Hz | 25 | 28 | 27 | 27 | 22 | 24 | 26 | 24 | 27 | 26 | 26 | 26 | 23 |
| 31.5Hz | 31 | 25 | 23 | 29 | 23 | 28 | 25 | 25 | 27 | 25 | 27 | 26 | 24 |
| 40Hz | 36 | 24 | 21 | 31 | 20 | 28 | 25 | 22 | 22 | 25 | 26 | 27 | 23 |
| 50Hz | 24 | 19 | 22 | 21 | 19 | 22 | 22 | 20 | 22 | 22 | 21 | 21 | 16 |
| 63Hz | 23 | 19 | 21 | 17 | 19 | 22 | 22 | 20 | 19 | 20 | 20 | 20 | 15 |
| 80Hz | 21 | 17 | 14 | 15 | 15 | 22 | 19 | 16 | 17 | 16 | 17 | 20 | 13 |
| 100Hz | 21 | 21 | 18 | 16 | 15 | 24 | 19 | 17 | 19 | 17 | 17 | 20 | 13 |
| 125Hz | 19 | 22 | 15 | 13 | 13 | 25 | 17 | 15 | 16 | 13 | 14 | 18 | 12 |
| 160Hz | 18 | 27 | 22 | 14 | 16 | 22 | 18 | 19 | 16 | 16 | 17 | 21 | 16 |
| 200Hz | 22 | 28 | 24 | 19 | 17 | 23 | 18 | 16 | 19 | 18 | 20 | 20 | 18 |
| 250Hz | 25 | 27 | 21 | 20 | 18 | 26 | 16 | 15 | 17 | 18 | 21 | 17 | 20 |
| 315Hz | 23 | 32 | 15 | 14 | 17 | 23 | 14 | 16 | 16 | 15 | 21 | 15 | 19 |
| 400Hz | 22 | 35 | 17 | 12 | 16 | 20 | 16 | 16 | 16 | 13 | 19 | 14 | 16 |
| 500Hz | 22 | 37 | 12 | 18 | 17 | 20 | 18 | 22 | 18 | 13 | 18 | 16 | 16 |
| 630Hz | 28 | 40 | 12 | 13 | 20 | 22 | 17 | 21 | 20 | 11 | 17 | 16 | 19 |
| 800Hz | 26 | 46 | 13 | 12 | 21 | 23 | 16 | 19 | 19 | 13 | 19 | 17 | 18 |
| 1kHz | 28 | 36 | 12 | 12 | 19 | 21 | 16 | 19 | 15 | 12 | 18 | 15 | 15 |
| 1.25kHz | 32 | 33 | 13 | 10 | 21 | 22 | 17 | 24 | 16 | 16 | 20 | 15 | 18 |
| 1.6kHz | 30 | 30 | 12 | 11 | 20 | 35 | 17 | 26 | 21 | 17 | 20 | 15 | 19 |
| 2kHz | 31 | 36 | 11 | 13 | 17 | 22 | 18 | 27 | 22 | 16 | 17 | 15 | 17 |
| 2.5kHz | 29 | 38 | 13 | 17 | 16 | 17 | 16 | 27 | 21 | 16 | 16 | 14 | 16 |
| 3.15kHz | 27 | 30 | 12 | 16 | 15 | 16 | 16 | 29 | 21 | 16 | 15 | 13 | 13 |
| 4kHz | 23 | 28 | 12 | 17 | 15 | 16 | 15 | 26 | 21 | 17 | 15 | 13 | 14 |
| 5kHz | 22 | 30 | 12 | 15 | 14 | 15 | 15 | 23 | 19 | 16 | 13 | 12 | 12 |
| 6.3kHz | 20 | 26 | 11 | 13 | 15 | 16 | 17 | 19 | 19 | 15 | 11 | 11 | 11 |
| 8kHz | 19 | 21 | 11 | 14 | 15 | 15 | 16 | 20 | 17 | 14 | 11 | 10 | 10 |
| 10kHz | 12 | 20 | 11 | 14 | 12 | 15 | 14 | 22 | 16 | 11 | 10 | 9 | 11 |
| 12.5kHz | 12 | 19 | 10 | 11 | 11 | 14 | 15 | 15 | 15 | 10 | 9 | 10 | 10 |
| 16kHz | 11 | 16 | 9 | 10 | 9 | 13 | 15 | 15 | 12 | 9 | 8 | 10 | 9 |
| 20kHz | 9 | 15 | 9 | 9 | 9 | 12 | 13 | 11 | 10 | 9 | 8 | 12 | 9 |
